# Nrf2 in Host Defense: Over the Rainbow

**DOI:** 10.1155/2013/975839

**Published:** 2013-10-27

**Authors:** Hye-Youn Cho, Mi-Kyoung Kwak, Jingbo Pi

**Affiliations:** ^1^Laboratory of Respiratory Biology, National Institute of Environmental Health Sciences, National Institutes of Health, 111 TW Alexander Drive, Building 101, MD D-201, Research Triangle Park, NC 27709, USA; ^2^The Catholic University of Korea, College of Pharmacy, 43 Jibong-ro, Wonmi-gu, Bucheon, Gyeonggi-Do 420-743, Republic of Korea; ^3^Institute for Chemical Safety Sciences, The Hamner Institutes for Health Sciences, Research Triangle Park, Durham, NC 27709, USA

Nuclear factor (erythroid-derived 2)-like 2 or NF-E2-related factor 2 (NRF2 or Nrf2 for rodents), was discovered about two decades ago and has been known as a key transcriptional regulator of antioxidant response element (ARE) bearing cytoprotective genes. Extensive investigation of NRF2 in host defense has been paralleled with increasing interests in oxidative stress that is a common occurrence in various critical disease conditions. A broad spectrum of NRF2 functions has been uncovered in clinical settings as well as in model diseases using gene-specific knockout mice or diverse cells and organs insulted by various oxidative stresses. Kelch-like ECH-associated protein 1 (KEAP1 or Keap1 for rodents) is a cytoplasmic suppressor of NRF2 and a critical modulator of NRF2 homeostasis and activity. Substantial discovery efforts into the molecular mechanisms of the KEAP1-NRF2 axis have found that stress-induced modifications of KEAP1 and NRF2 liberate NRF2 from the “hinge and latch”-like affinity binding of NRF2-KEAP1 [1], allowing nuclear accumulation and transactivation of NRF2. In normal physiologic conditions, however, NRF2 homeostasis is maintained by an ubiquitin ligase (Cullin 3-based E3 ligase) attached to the NRF2-bound KEAP1 homodimer, which tags NRF2 for proteasomal degradation. Interestingly, Nrf2 has been shown to induce specific catalytic subunits of the proteasome (e.g., murine Psmb5 and Psmb6 in the 20s proteasome) directly through ARE binding or indirectly [2]. While the lysosomal system is the principal mechanism for degrading proteins with long half-lives, the ubiquitin-proteasome system maintains protein quality by endoplasmic reticulum-associated degradation of misfolded or damaged proteins (e.g., oxidized proteins) as well as selective degradation of short-lived and regulatory proteins. In conjunction with the evidence of ARE-mediated NRF2 autoregulation [3, 4], it indicates that NRF2 homeostasis is maintained and adjusted by a tight regulatory circuit according to redox and proteolytic demands. 

NRF2 is known to subsidize host defense through involvement in complex pathways including thiol and other antioxidant activation as well as cell cycle and death, metabolism, immunity, selective protein degradation, development, and carcinogenesis. Potent phytochemical NRF2 agonists such as isothiocyanates including broccoli-originated sulforaphane have been widely applied to experimental systems to support the role for NRF2. In this special issue, fifteen research articles demonstrate molecular, cellular, and physiological aspects of NRF2- and ARE-mediated downstream mechanisms in various cells and tissues, many of which utilized gene knockout mice or their primary cells, NRF2 agonists or proteasome inhibitors, and *NRF2* or *KEAP1* gene silencing techniques. In addition, nine review articles highlight topics on recent advancements in NRF2 research, chemoprevention, immunity, metabolic disease, and genetic variation. 

NRF2 is highly expressed in organs such as liver, kidney, and lung that undergo routine detoxification processes. A number of contributions in this issue deal with the protective role of NRF2 in chemical-induced liver toxicity. A “graded Nrf2 activation model” is tested in mice by C. D. Klaassen and colleagues and elucidates an Nrf2 dose-dependent protection against various hepatotoxicants in mice. Y. Kumagai et al. summarize the findings of their own and by others on the S-mercuration of cellular proteins by methylmercury and the important protective role of the KEAP1/NRF2 pathway. S. I. Gum and Cho demonstrate the herbal medicine component ginsenoside (Rg3) as a potent inducer of Nrf2 and ARE-responsive multidrug resistance-associated proteins in the acetaminophen-induced hepatotoxicity model. M. K. Liu et al. suggest a coordinated nuclear export of BACH1 and nuclear accumulation of NRF2 in liver cells insulted by arsenic. A thorough review by Y. Shin et al. covers potential mechanisms and Nrf2 targets in various models of liver diseases including viral hepatitis and hepatocarcinomas in mice. 

Diabetes is one of the major causes of chronic kidney disease worldwide. One therapeutic approach to diabetic nephropathy is the use of compounds inducing cytoprotective genes as oxidative stress known to be implicated. Three articles by L. Cai and colleagues demonstrate that treatment with low-dose radiation (25 milligray daily for 3 days), sulforaphane (3 month), or a peptide aldehyde proteasome inhibitor (MG132) is effective in protection against diabetic nephropathy in mice, which is, at least in part, attributed to its action through Nrf2-ARE responses. Supporting these, murine Nrf2 has been known to have an essential role in metabolic disorders by controlling the capacity of adipose tissue expansion, insulin sensitivity, and glucose and lipid homeostasis [5]. It is also known that Nrf2 protects mice from high-fat-diet-induced obesity and insulin resistance. D. V. Chartoumpekis et al. profile hepatic transcriptome changes in high-fat-diet-induced obese mice. Their data ascertain that Nrf2 is involved in metabolic gene networks including bile acid synthesis from cholesterol, free fatty acid binding and transport, glucose metabolism, and glycerol transport in liver.

In murine heart, *Nrf2* deletion significantly increased susceptibility to environmental pollutants (ozone, particle) through changes in cardiac functions as demonstrated in an original research article by R. Howden et al. A beneficial contribution of Nrf2 in the heart transplantation model is suggested by K. C. Wu et al.: *Nrf2*-deficient (*Nrf2* ^−/−^) recipient mice did not support graft survival for longer than 7.5 days, while wild-type (*Nrf2* ^+/+^) hosts showed prolonged graft survival especially with sulforaphane treatment. Evidence suggests that NRF2 activity does not always lead to a positive outcome and may accelerate the pathogenesis of some cardiovascular diseases. For example, Nrf2 is found to promote atherosclerosis in animal models (e.g., apolipoprotein E mutant mice). Association of antioxidant defenses in cardiovascular diseases such as atherosclerosis, hypertension, heart failure, and ischemia-reperfusion injury is addressed in a review by R. Howden.

 The detrimental effects of NRF2 due to its aberrant activation have also been highlighted in recent years. Investigation in *Keap1*-deficient mice demonstrated uncontrolled accumulation of cytoplasmic Nrf2 by Keap1, which caused constitutive transactivation of Nrf2 and overproduction of downstream target genes leading to lethality associated with esophageal and forestomach hyperkeratosis [6]. Importantly, considerable investigations on biopsies of non-small-cell lung cancers (squamous cell carcinoma, large cell carcinoma, and adenocarcinoma) have indicated that frequent somatic (missense) mutations in *KEAP1* and/or *NRF2* are associated with persistent transactivation of NRF2 in metastatic cells. As a result, uncontrolled, overexpression of cytoprotective genes including drug efflux pumps is proposed to give selective growth advantage and chemoresistance of the metastatic cells. Comprehensive genome analyses also reported that the *NRF2*/*KEAP1*/*CUL3* pathway is one of the four most frequently mutated ones in human lung squamous cell carcinomas [7]. As discussed in a review by A. K. Bauer et al., these critical findings are controversial as to whether activation, or alternatively inhibition, of NRF2 is a strategy for the prevention or treatment of cancer. It implies that *KEAP1* may be a potential “tumor suppressor” gene, while *NRF2* may conversely be “oncogenic” in chemotherapy-resistant cancers, and effective personalized therapy may be warranted in patients with mutations. A research article by K.-A. Jung and M.-K. Kwak demonstrates a similar notion that *KEAP1* silencing enhances ARE-mediated aldo-keto reductase expressions and decreases cytotoxicity of colon cancer cells. A review by H.-Y. Cho profiles sequence variations including single nucleotide polymorphisms and somatic mutations discovered in human *NRF2* and murine *Nrf2* along with details on the genomic structure of *NRF2* and its homology to other vertebrate species. This review article compiles genetic and somatic mutations in association with disease risks including cancer metastases. 

While genetic and somatic mutations of *NRF2* are significantly relevant to the adverse consequences of neoplastic cells, preventative roles of Nrf2 toward experimentally-induced tumorigenesis have been demonstrated in diverse mouse tissues including colon, forestomach, gall bladder, and skin. E. Kobayashi et al. mention in a review that in the case of a tumor development model, Nrf2 controls phagocytosis, acute inflammation, and reactive oxygen species generation that are required for T cell suppression, whereby Nrf2 supports antitumor immunity and reduces tumor metastasis in myeloid-derived suppressor cells. The anticarcinogenic activity and specific targets of isothiocyanates are discussed by B. N. Das et al. in the current issue. Bioavailability of oral sulforaphane in various tissues has been determined recently [8], providing insights into its efficacy as a host-defense and chemopreventive agent. Increasing evidence indicates favorable roles for NRF2-ARE activation in neuronal disorders. C. Lee et al. demonstrate that *β*-amyloid peptide-induced brain cell apoptosis and toxicity is inhibited by sulforaphane, suggesting a potential intervention for Alzheimer's disease. R. Zhao et al. demonstrate in their paper a protective effect of another phytochemical antioxidant curcumin in keratinocytes against the cytotoxicity caused by a carcinogen inorganic arsenite. 

Airways are one of the most vulnerable tissues to oxidant injury. Protective roles for Nrf2 in nonmalignant lung disorders have been intensely studied [9], and two articles in this issue further support it: H. Y. Cho et al. demonstrate that *Nrf2* ^−/−^ mice have increased airway susceptibility to environmental ozone-induced inflammation and mucous cell metaplasia. H. R. Potteti et al. demonstrate that the Nrf2 pathway was not compromised but consistently functional during chronic hyperoxia exposure which induces airway epithelial cell death. This issue also adds a review article by X. Li et al. on Nrf2 in defense of allergic asthma caused by diesel exhaust particles present in urban air pollution. 

Novel information from advanced approaches of molecular genetics and computational modeling is reported in the current issue. M. R. Campbell et al. profile NRF2 target genes in sulforaphane-treated human lymphoblastoid cells by chromatin immunoprecipitation sequencing. Key pathways included hematopoiesis, which was further tested in erythroleukemic cells by silencing *NRF2* or *KEAP1* to support the role for Nrf2 in heme metabolism and erythropoiesis. T. Korcsmáros and colleagues introduce “NRF2-ome (http://nrf2.elte.hu/)” an integrated online resource and discovery tool for protein interaction and regulatory networks of NRF2. The authors extend the previously published NRF2 interactome and regulome [10], which lead to a total of 7,777 manually curated, integrated, and predicted interaction data for NRF2 along with its first neighbor interactors, target genes, regulating transcription factors, and microRNAs as well as NRF2 signaling pathways. 

We believe that the authors in the current issue add knowledge to the current understanding of NRF2-mediated molecular, cellular, and physiological mechanisms. We hope that readers of this NRF2 issue gain insights into potential therapeutic strategies of NRF2 agonists or antagonists in host defense and disease pathogenesis. 
